# Ovine *FABP4* Variation and Its Association With Flystrike Susceptibility

**DOI:** 10.3389/fgene.2021.675305

**Published:** 2021-06-15

**Authors:** L. E. R. Burrows, H. Zhou, C. M. A. Frampton, R. H. J. Forrest, J. G. H. Hickford

**Affiliations:** ^1^Gene-Marker Laboratory, Faculty of Agriculture and Life Sciences, Lincoln University, Christchurch, New Zealand; ^2^Department of Medicine, University of Otago, Christchurch, New Zealand; ^3^Faculty of Education Humanities and Health Science, Eastern Institute of Technology, Napier, New Zealand

**Keywords:** *FABP4*, flystrike, PCR-SSCP, variation, sheep

## Abstract

Flystrike is a major cost and a welfare issue for the New Zealand sheep industry. There are several factors that can predispose sheep to flystrike, such as having fleecerot, a urine-stained breech, and “dags” (an accumulation of fecal matter in the wool of the breech). The FABP4 gene (*FABP4*) has been associated with variation in ovine fleecerot resistance, with a strong genetic correlation existing between fleecerot and flystrike occurrence. In this study, blood samples were collected from sheep with and without flystrike for DNA typing. PCR-SSCP analyses were used to genotype two regions of ovine *FABP4*. Sheep with the *A*_1_ variant of *FABP4* were found to be less likely (odds ratio 0.689, *P* = 0.014) to have flystrike than those without *A*_1_. The likelihood of flystrike occurrence decreased as copy number of *A*_1_ increased (odds ratio 0.695, *P* = 0.006). This suggests that *FABP4* might be a candidate gene for flystrike resilience in sheep, although further research is required to verify this association.

## Introduction

The breeding of sheep that are less susceptible to flystrike is an attractive proposition for reducing its impact on sheep production. If genetic variation associated with decreased susceptibility to flystrike could be identified, then this might provide greater accuracy for making breeding selections to reduce the prevalence of the disease.

In 2010, a single-nucleotide polymorphism (SNP) association study was undertaken by Smith et al. (2010) on sheep from the fleecerot and flystrike resistant and susceptible flocks in Australia. Among the genes identified as potentially being associated with fleecerot susceptibility was the fatty-acid binding protein 4 (FABP4) gene (*FABP4*). Given that fleecerot has been identified as an important predisposing factor to flystrike (Norris et al., 2008) and that a study by Raadsma (1991) reported a strong genetic correlation (*r* > 0.9) between the two conditions, there is justification for testing whether *FABP4* is also associated with the occurrence of flystrike.

The fatty-acid binding proteins are hydrophobic ligand-binding cytoplasmic proteins, and are thought to be involved in lipid metabolism through the binding and intracellular transport of long-chain fatty-acids (Furuhashi and Hotamisligil, 2008; Tsuda et al., 2009). Expression of *FABP4* has been reported in adipocytes and macrophages (Furuhashi and Hotamisligil, 2008). In skin, FABP4 has been shown to be localized in the sebaceous glands (Watanabe et al., 1997), and it has been suggested to regulate their activity by modulating lipid-signaling and/or lipid metabolism in sebocytes (Lin and Khnykin, 2014).

Tsuda et al. (2009) revealed that *FABP4* was strongly expressed in phosphatase-and-tensin (*Pten*)-null keratinocytes. In this context, FABP4 has been suggested to selectively enhance the activities of peroxisome proliferator-activated receptor gamma (PPARγ), a member of the nuclear hormone receptor family, and one that regulates genes involved in sebaceous tissue differentiation (Michalik and Wahli, 2007). Keratinocyte-specific *Pten*-null mice display distinct phenotypes, which includes having wrinkled skin and ruffled and shaggy hair. Histological examination revealed that these mice had acanthosis, sebaceous gland hyperplasia, and accelerated hair follicle morphogenesis (Suzuki et al., 2003). The gene, *FABP4*, is therefore implicated in the development of these phenotypes. Given the loss of waxes and hydrophobicity is thought to be a major contributing factor in the development of fleecerot (Norris et al., 2008), then *FABP4* variation may also affect flystrike susceptibility in sheep. Furthermore, the FABP4 protein is also released from adipocytes and macrophages, and it is a proinflammatory signaling protein, with the level of FABP4 protein increasing in the blood of people with the skin inflammatory condition psoriasis (Baran et al., 2017). Inflammation is observed in skin tissues affected by fleece rot.

Yan et al. (2012) studied sequence variation in two regions of *FABP4*. In the first, which spanned parts of exon 2 and intron 2, five unique sequences were observed. These variants were a consequence of three nucleotide substitutions and one nucleotide deletion in intron 2. In the second region of the gene, which spanned parts of exon 3 and intron 3, four different sequences were detected, which come about as a consequence of four nucleotide substitutions.

To ascertain whether variation in *FABP4* is associated with variation in susceptibility to flystrike in New Zealand (NZ) sheep, animals with and without flystrike were identified at shearing time. Genetic variation within *FABP4* was analyzed using PCR-SSCP analyses, and associations with the occurrence of fleecerot and flystrike explored. Any association identified might provide a better understanding of biological role of *FABP4* in variation in susceptibility to flystrike.

## Materials and Methods

### Sheep Studied and Identification of Flystrike

Eight-hundred and fourty-four sheep were studied in total. Of these, 441 either had flystrike or were recovering from flystrike. Sheep were diagnosed as having active flystrike when larvae could be seen on the skin, and the wool was discolored and bad smelling. Sheep that had had flystrike were identified through having areas of pink skin with no wool or shorter wool, evidence of scar tissue from maggot damage, or flaky dry skin. Where possible, for each sheep found with flystrike, another sheep from the same flock, but that had no evidence of flystrike was also chosen (*n* = 403). The sheep were identified over a period of 5 years (2013–2017) from both commercial and stud farms from six geographical regions and were of 20 different breeds or composites there-of.

### Blood Collection

Blood samples from the sheep were collected onto FTA cards (Whatman, Middlesex, United Kingdom) and these were labeled with year, farm, breed, gender, age, and the presence (*n* = 441) or absence (*n* = 403) of flystrike as specified above. The blood was left to dry and then stored in darkness at room temperature. DNA stored in this way is suitable for subsequent analysis (Dash et al., 2020), and for genotyping the DNA was purified according to the method of Zhou et al. (2006).

### FABP4 Genotyping

The genotypes for Region-1 and -2 of *FABP4* were determined according to the methods described in Yan et al. (2012). Primers were synthesized by Integrated DNA Technologies (Coralville, IA, United States). All 844 sheep were genotyped for Region-1, while 582 were genotyped for Region 2 (*n* = 291 with flystrike).

### Statistical Analyses

All statistical analyses were performed using IBM SPSS Statistics version 24 (Chicago, IL, United States).

For both *FABP4* regions, the presence or absence of a sequence variant in each genotype was coded with a 1 or 0, respectively. For inclusion in statistical models, some variables and/or categories within variables were combined due to there being small numbers in some groups. Age and gender were merged into a single variable (age_gender) which had the following categories: animals under 2 years of age (gender was not known), 2 year-old ewes, 3 + year-old ewes, 2 year-old rams, and 3 + year-old rams. Sheep breed was compressed into four categories: black-face/terminal sheep (*n* = 173; Dorset Down + Finn x Texel Cross + Poll Dorset + Shropshire + South Down + South Suffolk + Texel + other Suffolk-crosses), Merino and Merino-cross sheep (*n* = 303; Merino + Corriedale + other Merino crosses), maternal cross-bred sheep (*n* = 236; Coopworth + Corriedale x Romney-cross + Perendale-cross + unspecified cross + Romney-cross + other composite maternal sheep) and pure-bred maternal sheep (*n* = 132; Romney + Perendale + Lincoln). The six geographical regions (Banks Peninsula, Mid-Canterbury, Northland, North Canterbury, Otago and South Canterbury) were combined into three general locations: mid-Canterbury, districts north of mid-Canterbury and districts south of mid-Canterbury.

Univariate Pearson Chi-square tests were then performed to explore the association between these variables: year (recognizing there is likely to be annual variation in flystrike occurrence), age_gender (recognizing there are likely to sheep age and gender effects on flystrike occurrence), breed (recognizing there may be breed effects in flystrike occurrence), and location (recognizing potential regional differences in flystrike occurrence), and the presence or absence of flystrike.

For each *FABP4* variant from the two regions of the gene, a Pearson Chi-squared test along with a binary logistic regression was performed to explore whether the presence or absence of the variant was associated with the presence or absence of flystrike, and to determine an odds-ratio (OR). If an association was detected, a subsequent binary logistic regression analysis was performed to determine the independent effects of the gene variants on the incidence of flystrike when year, breed, location, and age-gender were taken into account. A copy-number analysis was also performed in which the presence or absence of the variant was replaced with number of copies of the variant (0, 1, or 2) in the binary logistic regression models.

## Results

### Sequence Variation in FABP4

The previously described *A_1_, B_1_, C_1_* and *D*_1_ variants (exon 2—intron 2; Region-1) and *A_2_, B_2_*, and *C*_2_ variants (exon 3-intron 3; Region-2) of *FABP4* (Yan et al., 2012) were identified. The *E*_1_ (Region-1) and *D*_2_ (Region-2) variants (Yan et al., 2012) were not detected in the sheep studied here. The sequence variation in the regions typed could, in part, be matched to the findings of Smith et al. (2010) with the SNPs c.348 + 166T > C, c.348 + 298T > C, and c.348 + 356T > C in Region-2 of *FABP4*, corresponding to FABIn30227, FABIn30360, and FABIn30420 respectively, defined in that study.

### FABP4 Associations

The frequency of flystrike occurrence for sheep with different *FABP4* variants from each region of the gene, is shown in Table 1.

**TABLE 1:** Frequencies of flystrike in sheep with *FABP4* Region-1 and Region-2 variants.

Variant	Number of sheep with variant*	Number of sheep with variant and flystrike	Frequency of flystrike in animals with variant (%)
Region-1	(844 sheep in total)		
*A*_1_	314	143	46
*B*_1_	483	257	53
*C*_1_	521	284	55
*D*_1_	94	48	51
Region-2	(582 sheep in total)		
*A*_2_	489	246	50
*B*_2_	278	147	53
*C*_2_	19	9	47

*Includes heterozygous and homozygous sheep.

The results of the univariate analyses exploring the effect of the different independent effects on associations between the presence or absence of each *FABP4* Region-1 variant and flystrike presence and absence are shown in Table 2. Associations existed for each variable with at least one of the variants, and thus all the variables were retained in the subsequent multivariate analyses. The univariate Pearson chi-squared analyses to explore the association between the variables and the occurrence of flystrike detected no association for location (*P* = 0.545), year (*P* = 0.132), or breed (*P* = 0.050), but an association was detected with age_gender (*P* < 0.001). Only the association with age-gender persisted in the multivariate binary logistic models for each *FABP4* Region-1.

**TABLE 2:** *P*-values^a^ from Pearson Chi-square analyses exploring associations between the variables, each *FABP4* Region-1 variant and flystrike occurrence.

Variable	*FABP4* Region-1 variants				Flystrike
	*A*_1_	*B*_1_	*C*_1_	*D*_1_	
Age_gender	**0.001**	0.782	0.232	0.215	**<0.001**
Breed	**<0.001**	0.535	**0.004**	0.332	**0.050**
Location	0.584	0.406	**0.002**	0.635	0.545
Year	**0.001**	**<0.001**	**<0.001**	0.868	0.132

**^*a*^P ≤ 0.05 in bold.**

The presence of *FABP4 A*_1_ was found to be associated with a lower likelihood of flystrike (OR = 0.651, *P* = 0.003), which persisted when correcting for the effect of age_gender, breed, location and year (OR = 0.689, *P* = 0.014), while the presence of *C*_1_ appeared to possibly be linked to an increased likelihood of flystrike (OR = 1.267, *P* = 0.095) in the chi-square, but this was lost when corrected for the other effects (Table 3).

**TABLE 3:** The association of the presence of each *FABP4* Region-1 variant with the occurrence of flystrike given the presence of a particular *FABP4* Region-1 variant.

Statistical model	Variant	Odds ratio	95% confidence interval		*P*-value^a^
			Upper	Lower	
Pearson Chi-square, Binary logistic regression analysis: Dependent variable = the	*A*_1_	0.651	0.492	0.862	**0.003**
presence or absence of flystrike; independent variable = the presence or	*B*_1_	1.094	0.833	1.437	0.519
absence of the gene variant.	*C*_1_	1.267	0.959	1.673	0.095
	*D*_1_	0.948	0.617	1.456	0.807
Binary logistic regression: dependent variable = the presence or absence of	*A*_1_	0.689	0.512	0.927	**0.014**
flystrike; independent variables = the presence or absence of the gene variant, year,	*B*_1_	1.110	0.832	1.480	0.478
breed, location, and the combined age_gender variable.	*C*_1_	1.209	0.899	1.625	0.210
	*D*_1_	1.001	0.638	1.570	0.996

**^*a*^P ≤ 0.05 in bold.**

The copy number analysis (correcting for year, breed classification, location, and age-gender) for *FABP4 A_1_* revealed there was a 30.5% reduction in flystrike for each additional copy of *FABP4 A_1_* (OR = 0.695, *P* = 0.006).

No associations between the occurrence of flystrike and the variants of *FABP4* Region-2 were detected (Table 4).

**TABLE 4:** The association of the presence of each *FABP4* Region-2 variant with the occurrence of flystrike given the presence of a particular *FABP4* Region-2 variant.

Statistical model	Variant	Odds ratio	95% confidence interval		*P*-value
			Upper	Lower	
Pearson Chi-square, Binary logistic regression analysis: Dependent variable = the	*A*_2_	1.080	0.693	1.683	0.734
presence or absence of flystrike; independent variable = presence or absence of	*B*_2_	1.247	0.900	1.727	0.184
the gene variant.	*C*_2_	0.897	0.359	2.240	0.816
Binary logistic regression: dependent variable = the presence or absence of	*A*_2_	1.100	0.687	1.762	0.691
flystrike; independent variables = the presence or absence of the gene variant,	*B*_2_	1.161	0.814	1.655	0.409
year, breed, location, and the combined age_gender variable.	*C*_2_	1.075	0.402	2.872	0.886

## Discussion

Smith et al. (2010) identified five SNPs in ovine *FABP4* from Merino sheep that are associated with fleecerot. Three of these SNPs were also identified by Yan et al. (2012, 2018) and in this study too. In Region-1, Yan et al. (2012) detected five variant sequences of *FABP4* (named *A_1_ -E_1_*) and four variant sequences (*A_2_-D_2_*) for Region-2 in 483 NZ sheep of various breeds. Variants *A*_1_, *B*_1_ and *C*_1_ were the most common and observed in all breeds. Variant *D*_1_ was observed in all the breeds except Poll Dorset sheep and *E*_1_ was only observed in two breeds (Poll Dorset and Corriedale). In this study four of the *FABP4* Region-1 variants were detected (*A_1_-D_1_*), with *E*_1_ not being found. More recently, Yan et al. (2018) reported the five previously identified Region-1 variants (*A*_1_, *B*_1_, *C*_1_, *D*_1_ and *E*_1_) and three previously identified Region-2 variants (*A*_2_, *B*_2_ and *C*_2_) were detected in NZ Romney sheep, describing fourteen different haplotypes spanning the two regions: *A_1_-A_2_*, *A_1_-B_2_*, *A_1_-C_2_*, *B_1_-A_2_*, *C_1_-A_2_*, *C_1_-C_2_*, *D_1_-A_2_*, *B_1_-B_2_*, *C_1_-B_2_*, *D_1_-C_2_*, *E_1_-B_2_*, *E_1_-A_2_*, *D_1_-B_2_* and *B_1_-C_2_*.

In the context of the observed associations with Region-1 variant *A*_1_ in this study, it must, therefore, be noted that *A*_1_ could potentially be present in the sheep studied in one of three haplotypes across these two regions (*A_1_-A_2_*, *A_1_-B_2_*, and *A_1_-C_2_*), although the haplotypes were not established here as that require nucleotide sequencing of all 844 sheep. Variant *C*_1_ was also found in three haplotypes (*C_1_-A_2_*, *C_1_-C_2_*, and *C_1_-B_2_*) by Yan et al. (2018), albeit in different breeds. Variants *A*_2_ and *B*_2_ occurred in haplotypes with all five Region-1 variants, and *C*_2_ with all except *E*_1_ (Yan et al., 2018). It is therefore not surprising that associations were not detected with Region-2 of the gene and future research focused on association studies using haplotypes is merited.

The SNPs described in *FABP4* in this study were all found in introns. Although introns do not code for amino acids, some SNPs in introns, silent substitutions in coding sequences and variation in regions flanking coding sequences can have a functional role, and/or affect gene expression. For example, SNPs in introns have been reported to affect primary transcript splicing efficiency, the stability of mRNA, and the translation of mRNA (Le Hir et al., 2003); and silent substitutions in coding regions (typically in position three of the codon) have also been shown to affect mRNA stability and pre-mRNA splicing (Duan et al., 2003; Supek et al., 2014). The *FABP4* SNPs identified do not fall into any known RNA splicing sites, however, they may be linked to sequence variation in other regions of *FABP4* that is of structural or functional importance. This has also been suggested by Smith et al. (2010) and Yan et al. (2012, 2018).

Given that the aim of this study was to determine if there was an association between the presence of flystrike and variation in ovine *FABP4*, then it is notable that sheep with the *A*_1_ variant were less likely to have flystrike than those without *A*_1_. It is, however, impossible to say if any given sheep carrying the *A*_1_ variant will, or will not get flystrike, as some sheep in this study carrying *A*_1_ also had flystrike and some sheep carrying two copies of *A*_1_ were also struck, with them accounting for 2.5% of the struck genotypes. It must be remembered in this context that the analyses are only finding associations rather than causative mutations.

The first associations between SNPs in or near *FABP4*, and fleecerot, were described by Smith et al. (2010). However, given there is a high genetic correlation (*r* > 0.9) between fleecerot and flystrike, it is perhaps not that surprising that an association has been found with flystrike susceptibility. The susceptibility of sheep to fleecerot and flystrike can also be assessed through the measurement of indicator traits, including assessment of physical characteristics and chemical characteristics. In the context of the latter, wax and suint levels affect the wettability of the wool (Belschner, 1937; Norris et al., 2008), and thus susceptibility to disease. In fine wool sheep, bright (high luster), high yielding (but greasy) wool, which is white in color, is the most consistent wool type associated with fleecerot resistance, and wool of this type is highly heritable (Hayman, 1953; James et al., 1984; Raadsma, 1987). Fiber diameter is another highly heritable trait that has been associated with resistance to fleecerot (Raadsma, 1993), with a lower fiber diameter resulting in a greater resistance in Merino sheep (James and Ponzoni, 1992). Given these observations, the wax levels in the wool are possibly influenced by FABP4, and this could explain why variation in *FABP4* appears to be associated with fleecerot and flystrike resilience. In this context, exploring the relationship between *FABP4* expression and wool wax levels, along with other wool traits, could shed light on FABP4’s role in flystrike resilience, although it has been reported that the phenotypic correlations between fleecerot, flystrike and other fleece characteristics, are typically low (Raadsma, 1987).

Bakhtiarizadeh et al. (2013) used Lori-Bakhtiari and Zel sheep to investigate the differences in *FABP4* expression in fat-tail tissue and visceral adipose tissue. Their results suggested that the expression of *FABP4* was higher in the fat-tail of Lori-Bakhtiari sheep, than expression in either the fat-tail or visceral tissue of the Zel sheep. The higher level of expression of *FABP4* in the fat-tail tissue of Lori-Bakhtiari sheep was related to there being more fatty-acid transportation into the fat-tail compared with the Zel sheep. If fat is a factor that affects flystrike resilience in sheep, then increased expression of *FABP4*, leading to more fatty-acid transport into different areas of the body could be an underlying mechanism for the variation in resilience. It would be interesting to look at the frequency of *FABP4* variants *A_1_-E_1_* in Lori-Bakhtiari and Zel sheep to see if they have a higher frequency of the *A*_1_ variant, especially as this variant has also been found to occur at a high frequency in a line of sheep that were deliberately bred for increased fatness (Yan et al., 2012). The Lori-Bakhtiari and Zel sheep could also be investigated to ascertain their resilience to flystrike in the context of *FABP4* variation.

Yan et al. (2012) found a vast difference between fat and lean lines of sheep and variation in *FABP4*. Sheep carrying the *FABP4 A_1_* variant were predominantly in the fat line, while sheep carrying the *C*_1_ variant was associated with the lean line. In the present study, sheep with the *A*_1_ variant were the least likely to get flystrike. While the level of expression (if any) of this variant was not ascertained in either study, it might nevertheless speculated that sheep with a higher carcass fat content, may also have an increased or different lipid content in their wool. Wool wax contains fatty acids, and these coat the wool fibers and skin, where they inhibit bacterial growth by lowering the pH of the skin surface (Lambers et al., 2006). It might, therefore, be worthwhile, to investigate whether *FABP4* affects wool wax production (qualitatively and quantitatively) and whether this affects the likelihood of fleecerot and/or flystrike occurring. However, if the *FABP4 A*_1_ variant decreases flystrike susceptibility and increases the fat content in meat, then farmers would have to decide if they want to selectively breed sheep for both these traits. This could cause conflict with consumer demand for healthier sheep that produce leaner meat. In some markets, the current consumer demand is for leaner meat due to the common perception that fat is linked to obesity and cardiovascular disease (Volk, 2007; Pethick et al., 2011), and this demand is met by producing leaner slaughter animals on-farm (Pethick et al., 2011).

Fatty acid-binding protein 4 has also been shown to modulate inflammatory responses in macrophages (Furuhashi and Hotamisligil, 2008). In macrophages lacking FABP4 (*FABP4 -/-)* there are several signaling pathways that are suppressed, including the production of cytokines such as tumor-necrosis factor –α (TNF-α), interleukin 1 β (IL β) and IL6 (Makowski et al., 2005; Furuhashi and Hotamisligil, 2008). It is of interest that inflammatory cytokines and T cell-dependent cytokine IL-2 and IFN–γ are produced in the skin during flystrike (Bowles et al., 1994). FABP4 also acts to coordinate functional interactions between macrophages and adipocytes in the adipose tissue (Furuhashi and Hotamisligil, 2008). The adipocytes sit near the skin surface, thus these cells could play a role in the inflammatory response to flystrike in the skin. The “fatter” type sheep, as shown by Yan et al. (2012) to have a high frequency of *A*_1_ variant, may therefore have greater resilience to flystrike by having an improved inflammatory response.

While the results of this study suggest that variation in *FABP4* might be used as a gene-marker for flystrike resilience in sheep and that breeding for sheep that carry the *A*_1_ variant may decrease disease prevalence, the pleiotropic nature of the gene, including its potential effect on other important production traits, suggests considerably more research needs to be undertaken.

## Data Availability Statement

The raw data supporting the conclusions of this article will be made available by the authors, without undue reservation.

## Ethics Statement

Ethical review and approval was not required for the animal study because the research we undertook was retrospective/observational (not experimental), with the animals analyzed being located on private farms where they are part of commercial sheep farming operations. They were not part of experimental flocks, and the flocks were not structured, or created for experimental purposes. The blood collection approach we employed falls within the allowable practices specified in Section 7.5 Animal Identification of the Animal Welfare (Sheep and Beef Cattle) Code of Welfare 2010, which is a code of welfare issued under the Animal Welfare Act 1999 (New Zealand Government).

## Author Contributions

HZ and JH contributed to conception and design of the study. LB collected the data, organized the database, performed the experiments, and wrote the first draft of the manuscript. LB, CF, and RF performed the statistical analysis. HZ, RF, and JH contributed to the manuscript revision. All authors read and approved the submitted version.

## Conflict of Interest

The authors declare that the research was conducted in the absence of any commercial or financial relationships that could be construed as a potential conflict of interest.
